# Characterizing CDK8/19 Inhibitors through a NFκB-Dependent Cell-Based Assay

**DOI:** 10.3390/cells8101208

**Published:** 2019-10-06

**Authors:** Jing Li, Hao Ji, Donald C. Porter, Eugenia V. Broude, Igor B. Roninson, Mengqian Chen

**Affiliations:** 1 Department of Drug Discovery and Biomedical Sciences, University of South Carolina, Columbia, SC 29208, USA; jl13@email.sc.edu (J.L.); chene@cop.sc.edu (H.J.); broude@cop.sc.edu (E.V.B.); 2 Senex Biotechnology, Inc., Columbia, SC 29208, USA; porter@senexbio.com

**Keywords:** CDK8, CDK19, CDK inhibitors, NFκB, thienopyridines, cell-based assays

## Abstract

Cell-based assays for CDK8/19 inhibition are not easily defined, since there are no known cellular functions unique to these kinases. To solve this problem, we generated derivatives of 293 cells with CRISPR knockout of one or both of CDK8 and CDK19. Double knockout (dKO) of CDK8 and CDK19 together (but not individually) decreased the induction of transcription by NFκB (a CDK8/19-potentiated transcription factor) and abrogated the effect of CDK8/19 inhibitors on such induction. We generated wild type (WT) and dKO cell lines expressing luciferase from an NFκB-dependent promoter. Inhibitors selective for CDK8/19 over other CDKs decreased TNFα-induced luciferase expression in WT cells by ~80% with no effect on luciferase induction in dKO cells. In contrast, non-selective CDK inhibitors flavopiridol and dinaciclib and a CDK7/12/13 inhibitor THZ1 (but not CDK4/6 inhibitor palbociclib) suppressed luciferase induction in both WT and dKO cells, indicating a distinct role for other CDKs in the NFκB pathway. We used this assay to characterize a series of thienopyridines with in vitro bone anabolic activity, one of which was identified as a selective CDK8/19 inhibitor. Thienopyridines inhibited luciferase induction in the WT but not dKO cells and their IC_50_ values in the WT reporter assay showed near-perfect correlation (R^2^ = 0.98) with their reported activities in a bone anabolic activity assay, confirming that the latter function is mediated by CDK8/19 and validating our assay as a robust and quantitative method for CDK8/19 inhibition.

## 1. Introduction

Small-molecule inhibitors of the Mediator kinase CDK8 and its paralog CDK19 are being actively developed by different groups for therapeutic applications in various cancers and other chronic diseases [1]. The first CDK8/19 inhibitor has entered clinical trials in estrogen receptor-positive breast cancer (ClinicalTrials.gov Identifier: NCT03065010) and another inhibitor in acute myeloid leukemia (ClinicalTrials.gov Identifier: NCT04021368). Evaluation and optimization of such inhibitors requires quantitative, robust and selective cell-based assays for CDK8/19 inhibition. The development of such assays is complicated, however, by the lack of known cellular functions that are unique to CDK8/19. In particular, there are no known protein substrates that would be phosphorylated exclusively by CDK8/19 [2]. The most widely used phosphorylation substrate to assay CDK8/19 kinase activity is a transcription factor STAT1. CDK8/19 are indeed primarily responsible for INFγ-induced STAT1 phosphorylation at S727 [3], whereas basal STAT1 S727 phosphorylation is exerted not only by CDK8/19 but also by other kinases ([4] and our unpublished data). While quantitation of STAT1 S727 phosphorylation in INFγ-treated cells offers a suitable assay for CDK8/19 inhibition, immunoblotting-based measurements are fairly laborious and require careful normalization for total STAT1 signal (which is itself upregulated by INFγ).

Since CDK8/19 are transcriptional regulators [5], measurements of CDK8/19-regulated gene expression offer a relatively direct type of assay for CDK8/19 activity. Genes that are regulated by CDK8/19 inhibition in the absence of other treatments have been identified through transcriptomic studies and found to differ among cell lines [6,7,8,9,10,11]. Measuring the expression of a CDK8/19 regulated gene in a specific cell line can be used to characterize different CDK8/19 inhibitors but the usual methods for measuring gene expression, such as qPCR, are relatively laborious and expensive. More importantly, no genes are known to be regulated exclusively by CDK8/19, raising questions about CDK8/19 dependence of any observed effects.

CDK8/19 have been identified as co-regulators of various transcription factors [1,5], such as TCF/LEF/β-catenin [12,13], SMADs [14], HIF1A [7], ERα [8] and NFκB [15]. In the latter three cases, CDK8/19 were shown to act downstream of the corresponding transcription factors through C-terminal domain (CTD) phosphorylation of RNA polymerase II (Pol II), allowing Pol II to detach from the promoter and complete transcription of the genes that are newly activated by these transcription factors. Since promoter–reporter constructs for many transcription factors are available, it is possible to use such constructs to measure CDK8/19 inhibition through relatively simple and inexpensive reporter-based assays, such as the luciferase assay. However, no transcription factors are known to be co-regulated exclusively by CDK8/19, requiring controls for CDK8/19 specificity of the reporter assay. The latter issue also pertains to more indirect assays, such as inhibition of cell proliferation in CDK8/19-dependent leukemia cell lines [9,10].

In the present study, we have developed a selectivity control applicable to various cell-based assays for CDK8/19 inhibition, by generating derivatives of human embryonic kidney 293 cells with CRISPR-mediated knockout of CDK8 and CDK19. We then introduced a reporter construct expressing firefly luciferase from an NFκB-dependent consensus promoter into the wild-type (WT) 293 cells and into their derivative with a double knockout (dKO) of both CDK8 and CDK19. The WT reporter cells provided a sensitive and robust assay for CDK8/19 inhibition of NFκB-induced transcription; whereas, the matching dKO reporter cells offered a selectivity control for CDK8/19 dependence of the effects of tested inhibitors. Using this assay, we have characterized different CDK8/19-inhibiting small molecules, including a series of thienopyridines with in vitro bone anabolic activity [16], one of which has been recently identified as a selective CDK8/19 inhibitor [17], as well as several inhibitors of other CDKs. Our results demonstrate a striking correlation between the effects on CDK8/19 and bone anabolic activity and provide a comparison of the effects of CDK8/19 inhibitors and inhibitors of some other CDKs on NFκB activity.

## 2. Materials and Methods

### 2.1. Generation of 293 Derivatives with CRISPR-Mediated Knockout of CDK8 and CDK19

Human embryonic kidney 293 cells (ATCC CRL-1573) were cultured in DMEM (high-glucose) media supplemented with 10% fetal bovine serum (FBS) and penicillin–streptomycin–glutamine (1×) at 37 °C, and 5% CO_2_ culture conditions. The plasmid vector pSpCas9n(BB)-2A-GFP (PX461) [18] was obtained from Dr. Feng Zhang (distributed by Addgene, RRID: Addgene 48140). The PX461 vector expresses Cas9n (D10A nickase mutant), which allows for a more gene-specific CRISPR-mediated genomic modification than wild-type Cas9 nuclease. Annealed oligos encoding CDK8/CDK19-specific targeting sgRNA sequences (CDK8-sgRNA-A: TGCAGCCCTCGTATTCAAACAGG; CDK8-sgRNA-B: GTCACGTCTACAAAGCCAAGAGG; CDK19-sgRNA-A: CGCCTTGTAGACGTGACCGTAGG and CDK19-sgRNA-B: GCGGAAAGATGGGTAAGAGCAGG) were cloned into the PX461 vector via BbsI restriction sites to generate constructs PX461-CDK8-sgA, PX461-CDK8-sgB, PX461-CDK19-sgA and PX461-CDK19-sgB. 293 cells were transiently transfected with the gene-specific CRISPR constructs (sgA and sgB) and sorted for GFP-positive transfected cells 48 h after transfection using FACS Aria II (Becton-Dickinson). Single cell clones were expanded and evaluated for gene-specific knockout through both genomic DNA PCR/sequencing and western blot analysis to identify the CDK8-KO and CDK19-KO single-knockout 293 derivatives. The CDK8/19 double-knockout (dKO) derivative was established using the same protocol with CDK8-specifc PX461 constructs and CDK19-KO cells. 

### 2.2. Western Blot Analysis of 293-CDK8/19 Knockout Derivatives

Cells were plated in 60 mm plates at a density of 1 × 10^6^ cells per plate in regular culture media and cultured for 24 h. Then cells were treated with 1 μM senexin B or solvent control (0.1% DMSO, MilliporeSigma, St. Louis, MO, USA) for 3 h before lysing cells in 0.5 mL RIPA (radio immunoprecipitation assay) lysis buffer with 1× protease inhibitor cocktail. The protein concentration of extracts was determined using the DC (detergent-compatible) protein assay (Bio-Rad Laboratories). Protein (50 μg) was resolved on 4–12% Express-Plus polyacrylamide gels in Tris-MOPS (SDS) running buffer (GenScript, Piscataway, NJ, USA), transferred to the PVDF (polyvinylidene difluoride) membrane, blocked with 5% non-fat milk and incubated with primary antibodies: CDK8 (sc-1521, Santa Cruz Biotechnology, Dallas, TX, USA), CDK19 (HPA007053, MilliporeSigma) and GAPDH (sc-32233, Santa Cruz Biotechnology) followed by either anti-goat (sc-2020, Santa Cruz Biotechnology), anti-rabbit (NA934, GE Healthcare, Chicago, IL, USA) or anti-mouse (NXA931, GE Healthcare) secondary antibodies. Bands were visualized with Western Lighting Plus ECL (enhanced chemiluminescence) detection reagent (Perkin Elmer, Waltham, MA, USA) using ChemiDoc Touch™ (Bio-Rad Laboratories, Hercules, CA, USA). Images were analyzed using ImageLab software (Bio-Rad, Version 5.2.1 build 11). 

### 2.3. QPCR Analysis of 293-CDK8/19 Knockout Derivatives

Cells were seeded in 12-well plates at density of 3 × 10^5^ cells per well in regular culture media 24 h before treatment. Cells were first pre-treated with 1 μM senexin B or solvent control DMSO (0.1%) for 1 h and then treated with or without 10 ng/mL TNF-α for 2 h. Total RNA was extracted using RNAeasy Mini Kit (Qiagen, Germantown, MD, USA) and 1 µg of total RNA was used to generate cDNA using iScript cDNA synthesis kit (Bio-Rad). Gene expression was quantified using iTaq Universal SYBR green super mix on the CFX384 Real time system (Bio-Rad). The primers used for real-time PCR were: CXCL1-F, GAAAGCTTGCCTCAATCCTG; CXCL1-R, AACAGCCACCAGTGAGCTTC; IL8-F, TCCTGATTTCTGCAGCTCTGT; IL8-R, AAATTTGGGGTGGAAAGGTT; RPL13A-F, GGCCCAGCAGTACCTGTTTA and RPL13A-R, AGATGGCGGAGGTGCAG. 

### 2.4. Generation of NFκB-Dependent Reporter Cell Lines in WT and CDK8/19 dKO 293 Cells

The lentiviral construct pHAGE-NFkB-TA-LUC-UBC-dTomato was generated by Darrell Kotton [19] (Addgene plasmid # 49335; http://n2t.net/addgene:49335; RRID: Addgene_49335) and used for virus production as described previously [8]. Parental WT 293 cells or CDK8/19 dKO 293 cells were transduced with the lentivirus and dTomato-positive cells were sorted out using FACS Aria III. Single-cell clones were expanded and tested for luciferase reporter activities under TNFα- and/or senexin B-treated and untreated conditions with Bright-Glo Luciferase Assay System (Promega, Madison, WI, USA). The clones with robust TNFα-induced reporter activity (293-WT-NFKB-LUC#8 and 293-dKO-NFKB-LUC#2) were used in subsequent NFκB-dependent cell-based assays. 

### 2.5. NFκB-Dependent Cell-Based Assays

The assay was performed in white-bottom 96-well plates with 293-WT-NFKB-LUC#8 and 293-dKO-NFKB-LUC#2 cells. On day 1, cells were seeded in polyethyleneimine-coated 96-well plates at the density of 5 × 10^4^ cells per well. On day 2, cells were treated with 10 ng/mL recombinant human TNF-α (Z00404-50, GenScript, Piscataway, NJ, USA) plus serial dilutions of tested inhibitors ranging from 0.3 nM to 10 μM concentrations for 3 h before adding 4 μL luciferin solution (15 mg/mL potassium luciferin in PBS, Cat# LUCK-2G, GoldBio, St Louis, MO, USA) to measure luciferase activity. Chemiluminescence intensity of each well was measured with ChemiDoc Touch™ (Bio-Rad) and quantified using ImageLab software (Bio-Rad). The raw data were then further processed with GraphPad Prism 7.0 (GraphPad Software, San Diego, CA, USA) for curve-fitting and IC_50_ calculation. Among the inhibitors used for testing, senexin B was obtained from Senex Biotechnology; didehydro-cortistatin A (dCA) was a gift from Dr. Phil S. Baran (Scripps Research Institute); tosyl-L-phenylalanyl-chloromethane ketone (TPCK) and flavopiridol were obtained from Santa Cruz (sc-201297 and sc-202157); dinaciclib was from APExBio (Houston, TX, USA), THZ1 from MedChemExpress (Monmouth Junction, NJ, USA), bortezomib from MilliporeSigma and palbociclib from Selleck Chemicals (Houston, TX, USA) and thienopyridine derivatives (15u, 15n, 15q, 15u, 15v and 15w) were synthesized for Senex by Asinex (Moscow, Russia). 

## 3. Results

### 3.1. Generation of CDK8/19 Single- and Double-Knockout Derivatives and Evaluation of Effects of Target Knockout on NFκB Induction of Cytokine Genes

**Figure 1:**
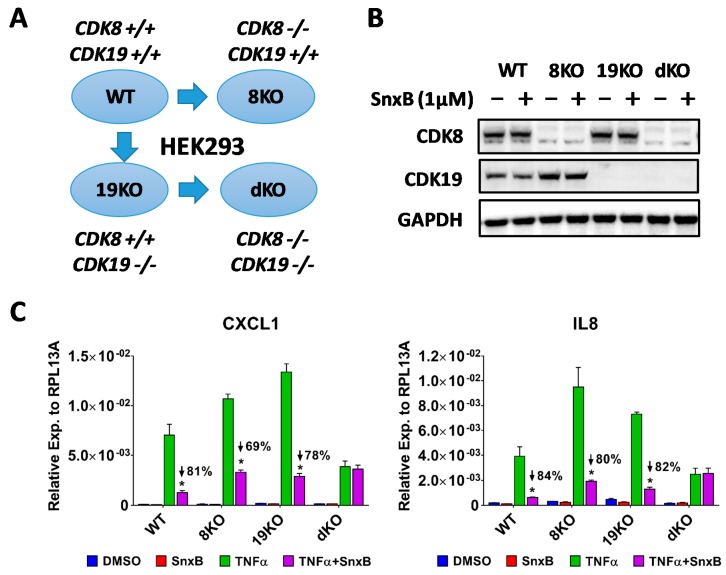
(**A**) Schematic of generating CDK8/19 single- and double-knockout derivatives of 293 cells. (**B**) Western blot analysis of CDK8 and CDK19 expression in 293 cells and their knockout derivatives in the absence or presence of senexin B (3 h treatment). (**C**) Effects of TNFα and senexin B on the mRNA expression of CXCL1 and IL8 in 293 cells and their knockout derivatives (QPCR). Data are presented as mean ± SEM (*n* = 3). Asterisks: *p* < 0.01 (*t*-test) for the differences between TNFα and TNFα + senexin B readouts.

To analyze different biological functions of CDK8 and CDK19, CRISPR technology was applied to generate CDK8 or CDK19 single knockout (8KO and 19KO, respectively) and CDK8/19 double knockout (dKO) derivatives of 293 cells (Figure 1A). 8KO and 19KO cells proliferated nearly as fast as parental wild-type (WT) 293 cells while dKO cells grew slower (33 h doubling-time of dKO vs. 24 h of WT; data not shown). Western blot analysis of these 293 derivatives treated with or without CDK8/19 inhibitor senexin B confirmed complete depletion of CDK8/19 proteins in single and double knockout and that CDK8/19 inhibition did not affect target protein expression (Figure 1B). 

In our previous study [15], we demonstrated that TNFα-induced NFκB-mediated transcriptional activation of acute inflammatory chemokines requires CDK8/19 kinase activity for maximal induction. We also showed that shRNA knockdown of CDK8 and CDK19 together decreases the induction and diminishes the inhibitory effects of CDK8/19 inhibitors [15]. Here by analyzing knockout derivatives of 293 cells (Figure 1C), we found that knockout of CDK8 or CDK19 alone did not interfere with the induction of CXCL1 and IL8 chemokines by TNFα (the differences between the WT and the single knockouts were within the range of clonal variability) but the knockout of both CDK8 and CDK19 (dKO) decreased chemokine induction. Notably, the magnitude of the inhibitory effect of senexin B was very similar among WT, 8KO and 19KO cells but this effect of CDK8/19 inhibition was completely abolished in dKO cells, providing a solid confirmation of our previous hypothesis that both CDK8 and CDK19 potentiate NFκB. 

### 3.2. Establishment and Validation of a NFκB Dependent Cell-Based Assay for CDK8/19 Inhibition

**Figure 2:**
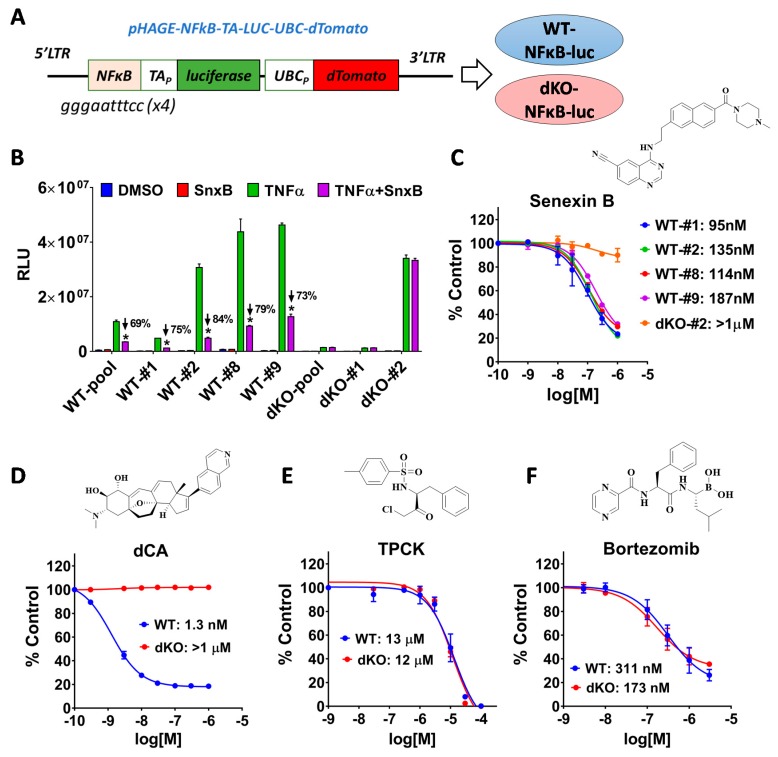
(**A**) Schematic of lentiviral construct pHAGE-NFkB-TA-LUC-UBC-dTomato. (**B**) Effects of treatment with 10 ng/mL TNFα, 1 μM senexin B or their combination on luciferase expression in the indicated pooled populations or clones of wild-type (WT) and double knockout (dKO) cells. Data are presented as mean ± SEM (*n* = 3). Asterisks: *p* < 0.01 for the difference between TNFα and TNFα + senexin B readouts. (**C**) Effects of different concentrations of senexin B on luciferase expression in the indicated WT and dKO 293 clones treated with 10 ng/mL TNFα for 3 h. % control (Y axis) was calculated relative to cells without the inhibitor. (**D**–**F**) Effects of different concentrations of dCA, TPCK and bortezomib on luciferase expression in 293-WT-NFKB-LUC#8 and 293-dKO-NFKB-LUC#2 reporter clones treated with 10 ng/mL TNFα for 3 h.

qPCR analysis, as in Figure 1C, is a sensitive but a rather laborious and expensive assay. To establish a more convenient cell-based assay for CDK8/19 inhibitors, we transduced the WT and dKO cells with a lentiviral construct that expresses firefly luciferase reporter gene from a consensus NFκB-dependent promoter that couples four repeats of a canonical NFκB-binding sequence with a minimal promoter (Figure 2A). The pooled population and four single-cell clones of construct-transduced WT cells, as well as the pooled population and two clones of dKO cells, were tested for TNFα-induced reporter activity and responses to CDK8/19 inhibition by CDK8/19 inhibitor senexin B [8]. In the WT cells 10 ng/mL of TNFα robustly induced luciferase expression and, to a lesser extent, in dKO cells but 1 μM senexin B inhibited this induction only in the WT but not in dKO cells (Figure 2B,C). The IC_50_ values for the inhibition of TNFα-induced luciferase expression by senexin B differed <two-fold among different WT clones (Figure 2C), with up to ~70–80% inhibition by 1 μM senexin B in all the clones (Figure 2B). This result indicates that CDK8/19-mediated potentiation of NFκB was not limited to specific genomic loci. We picked the clones with the highest levels of TNFα-induced reporter activity (293-WT-NFKB-LUC#8 and 293-dKO-NFKB-LUC#2) for our subsequent NFκB dependent cell-based assays. 

Figure 2D shows the effects of another, more potent CDK8/19 inhibitor, dCA (didehydro-Cortistatin A), an equipotent analog of cortistatin A [20] on TNFα-induced luciferase activity in these reporter cell lines. dCA had no effect on reporter induction in dKO cells but suppressed such induction in the WT reporter with IC_50_ of 1.3 nM (as compared to 114 nM for senexin B in the same cells). Maximal inhibition of the reporter induction by dCA reached a plateau at ~80%, similar to the maximal effect of senexin B. We also tested the effects of two widely used NFκB inhibitors, TPCK (tosyl-L-phenylalanyl-chloromethane ketone) that affects NFκB at concentrations >10 μM by inhibiting IKK [21] (Figure 2D) and proteasome inhibitor bortezomib active in sub-micromolar range (Figure 2E). Both TPCK and bortezomib inhibited the reporter activity in both WT and dKO with similar IC_50_ values, with the highest concentrations of TPCK achieving complete suppression of NFκB. 

### 3.3. Effects of Inhibitors of Other CDKs in the NFκB-Dependent Cell-Based Assay

**Figure 3:**
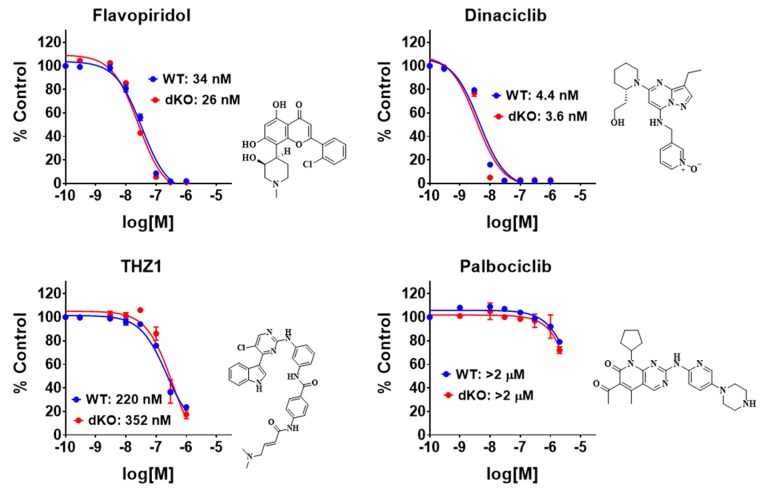
Effects of flavopiridol, dinaciclib, THZ1 and palbociclib at different concentrations on the induced NFκB reporter activity in WT and dKO 293 cells treated with 10 ng/mL TNFα for 3 h.

We further tested several inhibitors of other CDKs in the same assay. Flavopiridol (Alvociclib) is a potent inhibitor of multiple CDKs with preferential activity against CDK9, CDK4 and CDK7 [22]. Dinaciclib selectively inhibits cyclin dependent kinases CDK1, CDK2, CDK5 and CDK9 [23]. THZ1 inhibits CDK7, CDK12 and CDK13 [24] and palbociclib selectively inhibits CDK4 and CDK6 [25]. Flavopiridol, dinaciclib and THZ1 all completely inhibited NFκB-dependent promoter activation with indistinguishable IC_50_ values in WT and dKO cells, without the plateau typical for CDK8/19 inhibitors. In contrast, Palbociclib showed only weak inhibitory effects at high concentrations (>1 μM), in both WT and dKO cells (Figure 3). 

### 3.4. Analysis of a Series of Thienopyridine-Derivatives with Bone Anabolic Activity

**Figure 4:**
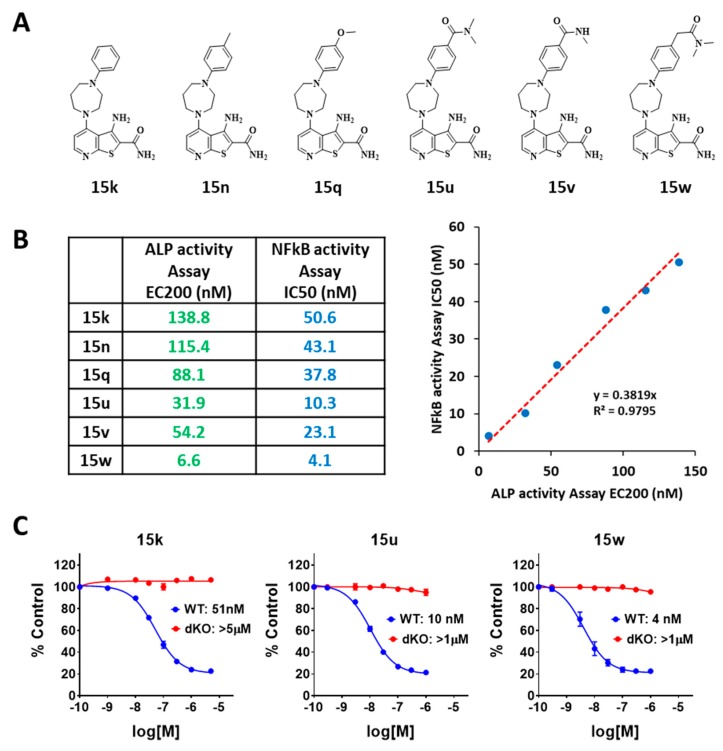
(**A**) Chemical structures of thienopyridine derivatives 15k, 15n, 15q, 15u, 15v and 15w. (**B**) Comparisons of IC_50_ values of the indicated thienopyridines measured in the NFκB reporter assay in WT 293 cells with the reported EC200 values in the ALPase activity assay in ST2 cells. (**C**) Effects of 15k, 15u and 15w at different concentrations on the induced NFκB reporter activity in WT and dKO 293 cells treated with 10 ng/mL TNFα for 3 h.

A recent publication reported that a thienopyridine derivative (15w) is a selective CDK8/19 inhibitor that (along with senexin B) promotes osteoblast mineralization and bone regeneration [17]. 15w is one of a series of compounds that were originally discovered and optimized for in vitro bone anabolic activity using an alkaline phosphatase (ALPase) activity assay in a mouse bone marrow stromal cell line ST2 [16]. To test if the activity of other compounds in the ALPase assay was associated with CDK8/19 inhibition, six thienopyridines with different ALPase-enhancing activities (15k, 15n, 15q, 15u, 15v and 15w) were synthesized and evaluated for CDK8/19 inhibitory activity in the NFκB-dependent cell-based assay (Figure 4A). All the thienopyridines exhibited strong inhibitory activities in the 293-WT-NFκB-Luc cell-based assay with IC_50_ values ranging from 4.1 nM to 50.6 nM and plateau inhibition of ~80% (Figure 4B). Interestingly, the IC_50_ values measured in this assay were very highly correlated with the values of EC200 (the concentration enhancing ALPase activity to 200% of the control) in the ALPase assay measured by Saito et al. [16] (R^2^ = 0.98), providing a strong indication that the in vitro bone anabolic activity is most likely mediated through CDK8/19 inhibition, in agreement with Amirhosseini et al. [17]. In addition, the inhibitory activities of 15k, 15u and 15w were also tested in 293-dKO-NFκB-Luc cells and none of them showed any activity in these cells (Figure 4C), demonstrating that NFκB inhibition by these compounds was mediated through CDK8/19.

## 4. Discussion

The fact that there are no known phenotypes affected exclusively by CDK8/19 presents a daunting problem for the development of cell-based assays for CDK8/19 inhibition. The approach described in this paper solved this problem by conducting phenotypic assays in both wild-type cells and in cells with the knockout of both CDK8 and CDK19. While implemented here for the NFκB-driven reporter system, this analysis should also be applicable to any other functional assays for CDK8/19. We note that using the dKO of both CDK8 and CDK19 was critical to this assay, as the knockouts of CDK8 or CDK19 had little effect on the response to the inhibitors, indicating that the two paralogs of the Mediator kinase could fully substitute for each other, at least in the case of NFκB.

The NFκB-driven luciferase reporter system described here provided a fast, inexpensive, robust and reproducible tool. Perhaps the best demonstration of the reliability of this assay appears in Figure 4C, where the IC_50_ values measured for six thienopyridine derivatives showed a near-perfect correlation with EC200 values that were determined by another group, in an entirely different assay, using different batches of the compounds. The results obtained in this analysis demonstrated CDK8/19-inhibitory activity for all six thienopyridines, most of which have not been previously tested for this activity, and it confirms the report that the bone anabolic activity is mediated by CDK8/19 inhibition [17]. The latter paper identified several factors affected by CDK8/19 inhibitors in bone marrow-derived cells and, remarkably, NFκB was one of these factors [17].

Comparison of the effects of CDK8/19 inhibitors with the inhibitors of other CDKs in the NFκB reporter assay demonstrated clear differences between the inhibitors of different CDKs. Two pleiotropic CDK inhibitors, flavopiridol and dinaciclib, and THZ1, a relatively specific inhibitor of CDK7/CDK12/CDK13, strongly inhibited NFκB activity in a CDK8/19-independent manner; whereas, CDK4/6 inhibitor palbociclib did not. Both flavopiridol [26] and dinaciclib [27] were previously shown to inhibit NFκB; in both cases, this effect was associated with decreased phosphorylation of IκBα, leading to decreased nuclear translocation of NFκB. It is unknown which CDK targets, if any, mediate the NFκB-inhibitory effects of these compounds, but one of their common targets, CDK9, was shown to interact with both p50 and p65 subunits of NFκB [28]. In the case of THZ1, one of its targets, CDK7, was shown to regulate NFκB by promoting its nuclear translocation [29]. Another THZ1 target, CDK12, was implicated in NFκB activation [30] but this effect was linked to the non-canonical NFκB activation pathway, which is not analyzed in our assay. Hence, CDK9 and CDK7 are likely to mediate the effects of other CDK inhibitors with NFκB-inhibitory activity. In contrast, inhibition of CDK4/6 and cyclin D was reported to have a complex effect on NFκB activity, at first stimulating NFκB nuclear translocation and then inhibiting NFκB activity [31,32]. However, the effects on the nuclear translocation of NFκB were not observed until 8-12 hrs of treatment [32], and it is not surprising therefore that the CDK4/6 inhibitor showed little effect on NFκB in our 3 h assay.

While CDK9 and CDK7 activate NFκB by promoting its nuclear translocation or binding to DNA, CDK8/19 act downstream of NFκB, via phosphorylation of the C-terminal domain of RNA polymerase II (Pol II) in the context of genes that have been newly activated by NFκB [15]. Remarkably, the inhibitors of other CDKs suppress TNFα-induced NFκB activity completely or nearly completely; whereas, all the tested CDK8/19 inhibitors inhibit it by no more than 80%, reaching a plateau at the highest concentrations. The same plateau was previously observed upon CDK8/19 inhibitor treatment in other NFκB reporter cell lines and in qPCR measurements of inhibitor effects [15]. The striking difference in the effects on NFκB activation between CDK8/19 inhibitors and the inhibitors of other CDKs illustrates the unique function of CDK8/19 as a gene context-specific downstream potentiator of NFκB and other transcription factors [15].

It is important to note that while the lack of an effect of a CDK8/19 inhibitor in dKO cells assures that the specific phenotypic effect (such as NFκB activation) measured in WT cells is mediated by CDK8/19, it provides no indication if the inhibitor has any off-target activities that do not impact the measured phenotype. For example, while dCA shows perfect CDK8/19 selectivity in the NFκB assay (Figure 2D), dCA, like cortistatin A, was found by kinome profiling to inhibit not only CDK8 and CDK19 but also ROCK1 and ROCK2 [6,33], an activity that is likely to account for a strong effect of cortistatin A/dCA in suppressing normal endothelial cell proliferation [34], a phenotype not shared by other CDK8/19 inhibitors [6]. Hence, kinome profiling and other off-target screening assays remain essential for the development of selective mediator kinase inhibitors.

## References

[1] Philip S., Kumarasiri M., Teo T., Yu M., Wang S. (2018). Cyclin-Dependent Kinase 8: A New Hope in Targeted Cancer Therapy?. J. Med. Chem..

[2] Poss Z.C., Ebmeier C.C., Odell A.T., Tangpeerachaikul A., Lee T., Pelish H.E., Shair M.D., Dowell R.D., Old W.M., Taatjes D.J. (2016). Identification of Mediator Kinase Substrates in Human Cells Using Cortistatin a and Quantitative Phosphoproteomics. Cell Rep..

[3] Bancerek J., Poss Z.C., Steinparzer I., Sedlyarov V., Pfaffenwimmer T., Mikulic I., Dolken L., Strobl B., Muller M., Taatjes D.J. (2013). Cdk8 Kinase Phosphorylates Transcription Factor Stat1 to Selectively Regulate the Interferon Response. Immunity.

[4] Staab J., Herrmann-Lingen C., Meyer T. (2013). Cdk8 as the Stat1 Serine 727 Kinase?. JAKSTAT.

[5] Fant C.B., Taatjes D.J. (2019). Regulatory Functions of the Mediator Kinases Cdk8 and Cdk19. Transcription.

[6] Porter D.C., Farmaki E., Altilia S., Schools G.P., West D.K., Chen M., Chang B.D., Puzyrev A.T., Lim C.U., Rokow-Kittell R. (2012). Cyclin-Dependent Kinase 8 Mediates Chemotherapy-Induced Tumor-Promoting Paracrine Activities. Proc. Natl. Acad. Sci. USA.

[7] Galbraith M.D., Allen M.A., Bensard C.L., Wang X., Schwinn M.K., Qin B., Long H.W., Daniels D.L., Hahn W.C., Dowell R.D. (2013). Hif1a Employs Cdk8-Mediator to Stimulate Rnapii Elongation in Response to Hypoxia. Cell.

[8] McDermott M.S., Chumanevich A.A., Lim C.U., Liang J., Chen M., Altilia S., Oliver D., Rae J.M., Shtutman M., Kiaris H. (2017). Inhibition of Cdk8 Mediator Kinase Suppresses Estrogen Dependent Transcription and the Growth of Estrogen Receptor Positive Breast Cancer. Oncotarget.

[9] Pelish H.E., Liau B.B., Nitulescu I.I., Tangpeerachaikul A., Poss Z.C., Da Silva D.H., Caruso B.T., Arefolov A., Fadeyi O., Christie A.L. (2015). Mediator Kinase Inhibition Further Activates Super-Enhancer-Associated Genes in Aml. Nature.

[10] Rzymski T., Mikula M., Zylkiewicz E., Dreas A., Wiklik K., Golas A., Wojcik K., Masiejczyk M., Wrobel A., Dolata I. (2017). Sel120-34a Is a Novel Cdk8 Inhibitor Active in Aml Cells with High Levels of Serine Phosphorylation of Stat1 and Stat5 Transactivation Domains. Oncotarget.

[11] Liang J., Chen M., Hughes D., Chumanevich A.A., Altilia S., Kaza V., Lim C.U., Kiaris H., Mythreye K., Pena M.M. (2018). Cdk8 Selectively Promotes the Growth of Colon Cancer Metastases in the Liver by Regulating Gene Expression of Timp3 and Matrix Metalloproteinases. Cancer Res..

[12] Firestein R., Bass A.J., Kim S.Y., Dunn I.F., Silver S.J., Guney I., Freed E., Ligon A.H., Vena N., Ogino S. (2008). Cdk8 Is a Colorectal Cancer Oncogene That Regulates Beta-Catenin Activity. Nature.

[13] Morris E.J., Ji J.Y., Yang F., Di Stefano L., Herr A., Moon N.S., Kwon E.J., Haigis K.M., Naar A.M., Dyson N.J. (2008). E2f1 Represses Beta-Catenin Transcription and Is Antagonized by Both Prb and Cdk8. Nature.

[14] Alarcon C., Zaromytidou A.I., Xi Q., Gao S., Yu J., Fujisawa S., Barlas A., Miller A.N., Manova-Todorova K., Macias M.J. (2009). Nuclear Cdks Drive Smad Transcriptional Activation and Turnover in Bmp and Tgf-Beta Pathways. Cell.

[15] Chen M., Liang J., Ji H., Yang Z., Altilia S., Hu B., Schronce A., McDermott M.S.J., Schools G.P., Lim C.U. (2017). Cdk8/19 Mediator Kinases Potentiate Induction of Transcription by Nfkappab. Proc. Natl. Acad. Sci. USA.

[16] Saito K., Nakao A., Shinozuka T., Shimada K., Matsui S., Oizumi K., Yano K., Ohata K., Nakai D., Nagai Y. (2013). Discovery and Structure-Activity Relationship of Thienopyridine Derivatives as Bone Anabolic Agents. Bioorg. Med. Chem..

[17] Amirhosseini M., Bernhardsson M., Lang P., Andersson G., Flygare J., Fahlgren A. (2019). Cyclin-Dependent Kinase 8/19 Inhibition Suppresses Osteoclastogenesis by Downregulating Rank and Promotes Osteoblast Mineralization and Cancellous Bone Healing. J. Cell Physiol..

[18] Ran F.A., Hsu P.D., Wright J., Agarwala V., Scott D.A., Zhang F. (2013). Genome Engineering Using the Crispr-Cas9 System. Nat. Protoc..

[19] Wilson A.A., Kwok L.W., Porter E.L., Payne J.G., McElroy G.S., Ohle S.J., Greenhill S.R., Blahna M.T., Yamamoto K., Jean J.C. (2013). Lentiviral Delivery of Rnai for in Vivo Lineage-Specific Modulation of Gene Expression in Mouse Lung Macrophages. Mol. Ther..

[20] Shi J., Manolikakes G., Yeh C.H., Guerrero C.A., Shenvi R.A., Shigehisa H., Baran P.S. (2011). Scalable Synthesis of Cortistatin a and Related Structures. J. Am. Chem. Soc..

[21] Ha K.H., Byun M.S., Choi J., Jeong J., Lee K.J., Jue D.M. (2009). N-Tosyl-L-Phenylalanine Chloromethyl Ketone Inhibits Nf-Kappab Activation by Blocking Specific Cysteine Residues of Ikappab Kinase Beta and P65/Rela. Biochemistry.

[22] Zeidner J.F., Karp J.E. (2015). Clinical Activity of Alvocidib (Flavopiridol) in Acute Myeloid Leukemia. Leuk. Res..

[23] Criscitiello C., Viale G., Esposito A., Curiglia G. (2014). Dinaciclib for the Treatment of Breast Cancer. Expert Opin. Investig. Drugs.

[24] Zeng M., Kwiatkowski N.P., Zhang T., Nabet B., Xu M., Liang Y., Quan C., Wang J., Hao M., Palakurthi S. (2018). Targeting Myc Dependency in Ovarian Cancer through Inhibition of Cdk7 and Cdk12/13. eLife.

[25] Fry D.W., Harvey P.J., Keller P.R., Elliott W.L., Meade M., Trachet E., Albassam M., Zheng X., Leopold W.R., Pryer N.K. (2004). Specific Inhibition of Cyclin-Dependent Kinase 4/6 by Pd 0332991 and Associated Antitumor Activity in Human Tumor Xenografts. Mol. Cancer Ther..

[26] Takada Y., Aggarwal B.B. (2004). Flavopiridol Inhibits Nf-Kappab Activation Induced by Various Carcinogens and Inflammatory Agents through Inhibition of Ikappabalpha Kinase and P65 Phosphorylation: Abrogation of Cyclin D1, Cyclooxygenase-2, and Matrix Metalloprotease-9. J. Biol. Chem..

[27] Chen Y., Germano S., Clements C., Samuel J., Shelmani G., Jayne S., Dyer M.J., Macip S. (2016). Pro-Survival Signal Inhibition by Cdk Inhibitor Dinaciclib in Chronic Lymphocytic Leukaemia. Br. J. Haematol..

[28] Amini S., Clavo A., Nadraga Y., Giordano A., Khalili K., Sawaya B.E. (2002). Interplay between Cdk9 and Nf-Kappab Factors Determines the Level of Hiv-1 Gene Transcription in Astrocytic Cells. Oncogene.

[29] Hong H., Zeng Y., Jian W., Li L., Lin L., Mo Y., Liu M., Fang S., Xia Y. (2018). Cdk7 Inhibition Suppresses Rheumatoid Arthritis Inflammation Via Blockage of Nf-Kappab Activation and Il-1beta/Il-6 Secretion. J. Cell Mol. Med..

[30] Henry K.L., Kellner D., Bajrami B., Anderson J.E., Beyna M., Bhisetti G., Cameron T., Capacci A.G., Bertolotti-Ciarlet A., Feng J. (2018). Cdk12-Mediated Transcriptional Regulation of Noncanonical Nf-Kappab Components Is Essential for Signaling. Sci. Signal..

[31] Thoms H.C., Dunlop M.G., Stark L.A. (2007). P38-Mediated Inactivation of Cyclin D1/Cyclin-Dependent Kinase 4 Stimulates Nucleolar Translocation of Rela and Apoptosis in Colorectal Cancer Cells. Cancer Res..

[32] Thoms H.C., Dunlop M.G., Stark L.A. (2007). Cdk4 Inhibitors and Apoptosis: A Novel Mechanism Requiring Nucleolar Targeting of Rela. Cell Cycle.

[33] Cee V.J., Chen D.Y., Lee M.R., Nicolaou K.C. (2009). Cortistatin a Is a High-Affinity Ligand of Protein Kinases Rock, Cdk8, and Cdk11. Angew. Chem. Int. Ed..

[34] Aoki S., Watanabe Y., Sanagawa M., Setiawan A., Kotoku N., Kobayashi M. (2006). Cortistatins a, B, C, and D, Anti-Angiogenic Steroidal Alkaloids, from the Marine Sponge Corticium Simplex. J. Am. Chem. Soc..

